# The NM_033380.2 transcript of the *COL4A5* gene contains a variable splice site c.4822–10T>C, which has been identified as a causative factor for Alport syndrome

**DOI:** 10.3389/fgene.2024.1330525

**Published:** 2024-05-16

**Authors:** Lei Liang, Haotian Wu, Jianrong Zhao

**Affiliations:** ^1^ Center for Prenatal Diagnosis and Medical Genetics, Affiliated Hospital of Inner Mongolia Medical University, Hohhot, China; ^2^ School of Public Health, Inner Mongolia Medical University, Hohhot, China; ^3^ Department of Nephrology, Affiliated Hospital of Inner Mongolia Medical University, Hohhot, China

**Keywords:** Alport syndrome, COL4A5, splicing variant, minigene assay, molecular dynamics

## Abstract

Alport Syndrome (AS) is a genetic kidney disorder characterized by progressive hearing loss and atypical eye symptoms, resulting in a poor prognosis and lack of effective targeted therapy. The primary mode of inheritance is X-linked dominant (XLAS) due to variants in the *COL4A5* gene. This study revealed a previously unidentified alternative form of the *COL4A5* gene, namely, the c.4822–10T>C variant, which was confirmed through *in vitro* experiments. To investigate the impact of a splicing variant on *COL4A5* mRNA production, an *in vitro* minigene splicing assay was utilized. Additionally, molecular dynamics was employed to predict the ability of α5(IV) to form a triple helix. Results from the experiment revealed that the wild-type (WT) plasmid produced two distinct mRNA products simultaneously. Sequence analysis using the BLAST database revealed a 173-bp deletion in the mRNA sequence of the first product, indicating a potential similarity to the XM_016942897.2 transcript of *Pan troglodytes*. The second mRNA product of the WT plasmid contained the full sequence of exons 51, 52, and 53, as anticipated. Conversely, the mutant (MT) plasmid generated a single mRNA product with a 173-bp deletion in exon 52, leading to the identification of the mature mRNA expression as NM_033380.2: COL4A5: c.4822_4994del. In the context of nonsense-mediated mRNA decay (NMD), the deletion c.4822_4994 results in the production of a truncated protein, p.His1608*, that terminates prematurely. This truncated protein may disrupt the secondary structure of α5(IV) and potentially cause an abnormal conformation of α345(IV). This study examines the relationship between the variable splicing pattern in the NM_033380.2 transcript of the *COL4A5* gene in XLAS patients and the presence of the *COL4A5* gene splice variant c.4822–10T>C. Our findings indicate that the c.4822–10T>C splice variant leads to activation of nonsense-mediated mRNA degradation (NMD) and reduced *COL4A5* mRNA expression, resulting in inadequate synthesis of the corresponding proteins. This aligns with the patient’s immunofluorescence results showing negative α5(IV) chain presence at the glomerular basement membrane, bursa, and tubular basement membrane, confirming the pathogenic nature of c.4822–10T>C.

## 1 Introduction

Alport syndrome (AS) is an inherited condition marked by the gradual deterioration of the kidneys, hearing loss, and abnormalities in the eyes (Nozu et al., 2019). AS is caused by a variant in one of the genes *COL4A3*, *COL4A4*, or *COL4A5*, which encode the α3-α5 chains of type IV collagen (Col-IV). There are three subtypes of AS, which are categorized according to the way it is inherited: X-linked (XLAS), autosomal recessive (ARAS), and autosomal dominant (ADAS). More precisely, XLAS is the result of a variant in the *COL4A5* gene, which is responsible for producing α5(IV). With the progression of sequencing technology, an increasing number of variants that may result in anomalous splicing have been uncovered (Groopman et al., 2019; Yamamura et al., 2019). Validating the underlying pathogenic mechanism is essential for determining the pathogenicity of these variants, but it requires *in vitro* experimental data (Nozu et al., 2014; Gao et al., 2020). Therefore, we propose that the examination of functional analysis, such as the minigene splicing assay, can help clarify the disease-causing potential of splicing variants that have uncertain significance. By using the minigene splicing assay, it becomes possible to assess the effects of changes on splicing mechanisms without requiring a sample from the patient’s cells. The purpose of this study was to assess the accuracy and possible outcomes of a newly identified splicing variant in the *COL4A5* gene. We explained the techniques that can detect the harmfulness of splicing variants that could lead to AS. Additionally, we presented a new method to predict the impact of the variants in the *COL4A5* gene on α345(IV) trimerization using molecular dynamics.

## 2 Materials and methods

### 2.1 Patient details

The individual is a preschool boy of 5 years old who experienced continuous visible blood in the urine for a duration exceeding 6 weeks. The onset of hematuria was insidious, and no obvious cause was identified. The patient’s condition deteriorated gradually, and there were no notable abnormalities observed in the blood count, biochemistry, or sedimentation rate. Nevertheless, the urine analysis revealed a high concentration of erythrocytes. The individual is of Han ethnicity and comes from Hohhot, Inner Mongolia Autonomous Region in mainland China. There was no previous occurrence of kidney disease in his family. The phenotypes of the patient’s parents are within the normal range.

### 2.2 Sanger sequencing and whole exome sequencing

The patient and his mother’s peripheral blood was obtained using an anticoagulant tube containing EDTA, and then genomic DNA was extracted using the QIAamp DNA Blood Mini Kit (Qigen, Hilden, Germany). The Human Comprehensive Exome Panel (Twist Bioscience, CA) was utilized for library preparation and exome capture, respectively. The captured libraries were subsequently sequenced on MGISEQ-T7 sequencer (BGI, Shenzhen, China).

FASTQC was used to assess the quality of the raw data. Afterwards, the unsoiled readings were aligned to the reference genome (GRCh37/hg19) utilizing BWA program. After removing duplicates and recalibrating base quality, the GATK pipeline was utilized to identify SNP and Indel variants. ANNOVAR was used to annotate the identified variations, which were then filtered based on minor allele frequencies (MAFs) below 0.5% in dbSNP, 1,000G, ExAC, and gnomAD databases.

Sanger sequencing was carried out on the patient’s blood DNA to verify the existence of the variant. The interpretation of sequence variation data follows the genetic variation classification standards and guidelines set by the American College of Medical Genetics and Genomics (ACMG), ClinGen sequence variation interpretation (SVI), and specific gene and disease guidelines. The detected variant underwent variable splicing analysis through the utilization of dbscSNV v1.1 software and SpliceAI (https://spliceailookup.broadinstitute.org/).

### 2.3 *In vitro* splicing validation experiments of mRNA of *COL4A5* gene

The *COL4A5* c.4822–10T>C variant was used to create both WT and MT plasmids. We utilized the pMini-CopGFP cloning vector containing BamHI/XhoI restriction sites. Both normal genomic DNA and genomic DNA containing the *COL4A5* c.4822–10T>C variant underwent amplification of seamless primers. Both the WT and MT segments of the desired gene were obtained and incorporated into the cloning vector using a dual enzyme digestion and recombination process. Afterwards, the resultant recombinant product was introduced into capable cells and cultivated, while clones were chosen for PCR amplification. Sanger sequencing was then conducted to verify the proper insertion of the *COL4A5* gene’s target fragment into the vector. Ultimately, the suitable recombinant plasmids for both the WT and MT minigenes were selected. Following the cultivation of a bacterial solution ranging from 5 to 15 mL, the plasmid was isolated and then introduced into 293 T cells through transfection. cDNA was extracted from the transfected cells after reverse transcription of RNA, and then primers were designed for RT-PCR amplification and gel electrophoresis.

### 2.4 Analyzing the conservation of amino acid residues and predicting the structure of mutated proteins through evolutionary studies

Protein sequences from different organisms were acquired from NCBI, and then analyzed for conservation using Jalview software through multiple sequence alignment. Transcript produced by the *COL4A5*:c.4822–10T>C variant were subjected to comparison with the Nucleotide collection (nr/nt) database through NCBI blast. The NM_033380.2 transcript and XM_016942897.2 transcript were further compared with transcripts for differential analysis at both the transcript and amino acid levels using SnapGene v6.0.2.

### 2.5 3-D structure analysis of α5(IV) and α345(IV)

Using the MODELLER software, we employed homology modeling to generate the 3-D structure of the WT-α345(IV) trimer. The triple helical region and the NC1 domains of α3(IV)–α5(IV) were modeled based on PDB IDs 3HQV, 5NB0, 5NB1, and 5NAZ. GROMACS version 2020.4 was utilized to optimize the structures of the WT-α5(IV) amino acid sequences, resulting in the preparation of the c.4822–10T>C MT-α5(IV) and its trimer’s 3-D structure. For all simulations, the AMBER99SB force field was utilized.

### 2.6 Molecular dynamics

The protein molecular dynamics were simulated using the GROMACS (GROningen Machine) software package. The protein employs the AMBER14sb (assisted model building with energyre finement) force field. The GROMACS module received the protein, along with the addition of hydrogen atoms and NaCl ions. Choose the prevailing TIP3P (transferable interatomic potential with three points model) water model and establish periodic boundary conditions. The molecular dynamics simulation workflow consists of four stages: energy minimization, NVT (canonical ensemble) equilibrium, NPT (constant-pressure, constant-temperature) equilibrium, and production dynamics simulation. Initially, the heavy atoms of the protein were restricted to reduce the energy of water molecules through 10,000 steps, which included 5,000 steps of the steepest descent method and 5,000 steps of the con-jugate gradient method. Subsequently, while keeping the constraints, a 50,000 step NVT ensemble simulation was performed for the entire system. The system was initially at a temperature of 298K and a time step of 2 fs. Subsequently, a 50,000 step NPT ensemble simulation was conducted for the entire system, maintaining the temperature at 298 K and the time step at 2 fs. Finally, a molecular dynamics simulation of the system was performed in the NPT ensemble for 100 ns, using a time step of 2 fs. The GROMACS software package module analyzed the pertinent parameters.

## 3 Results

### 3.1 Pathologic diagnosis

**FIGURE 1:**
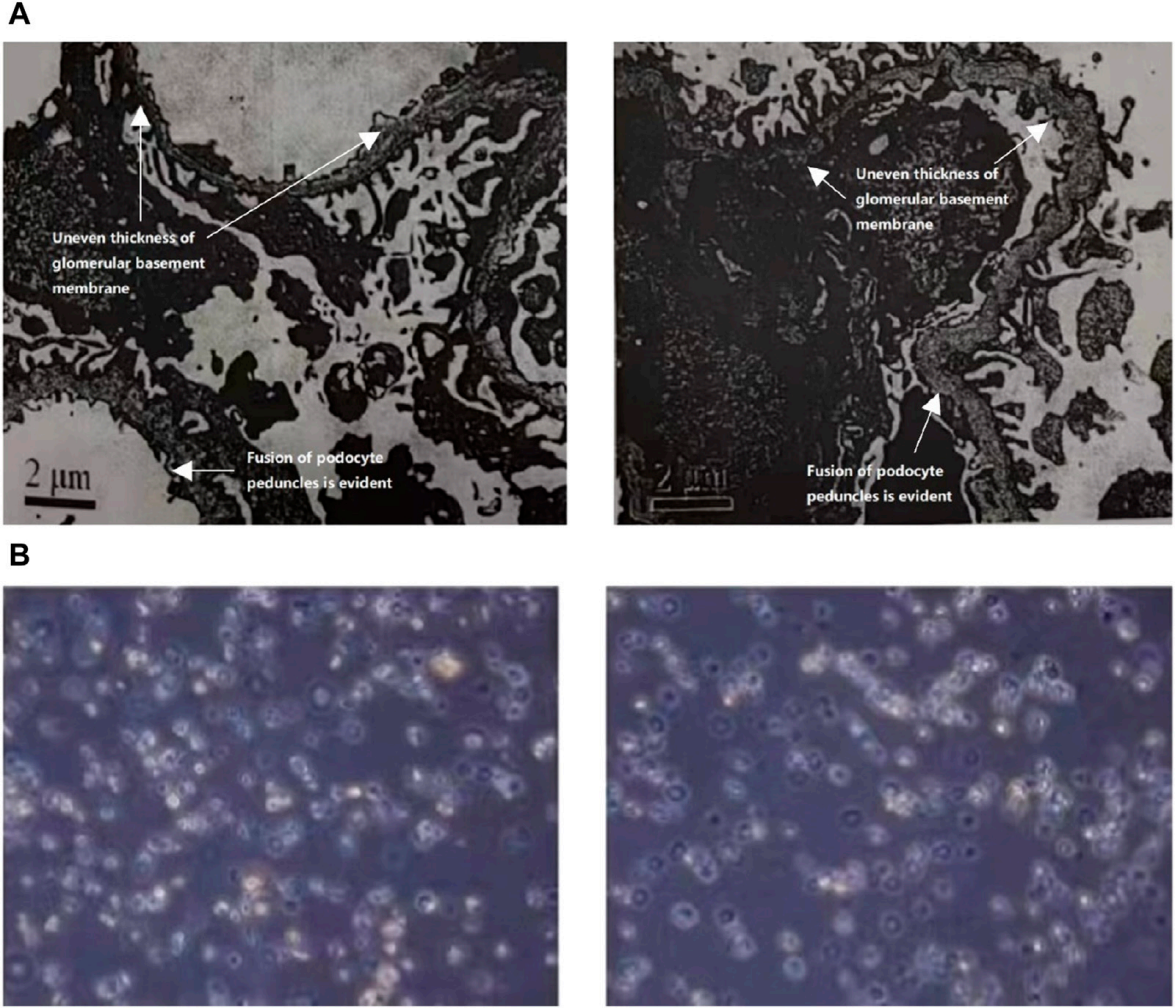
**(A)** The ultrastructural analysis of the kidney tissue from the patient. The electron microscope analysis demonstrated a notable increase in the population of glomerular mesangial cells and matrix, accompanied by an irregular thickening of the basement membrane, stratified dense layer, and insect-like alterations. Additionally, a significant fusion of epithelial foot processes was observed. **(B)** Urine red blood cell analysis of the patient. Urine red blood cell analysis revealed the presence of annular, crinkled, and anomalous erythrocytes in over 90.0% of full-field views.

In 2021, the urine protein/creatinine ratio of the child was 1.06 g/gcr, suggesting an elevated amount of protein in the urine compared to creatinine. The urine microalbumin level was 194.00 mg/L, which is also indicative of kidney damage. Additionally, the urine transferrin level was 15.60 mg/L, further suggesting kidney dysfunction. The measurement of protein in the urine over a 24-h period was confirmed to be 0.63 g/24 h through 24-h urine protein quantification. Upon light microscopy examination, it was noted that the renal puncture tissue had 25 glomeruli. These glomeruli displayed slight segmental proliferation of glomerular thylakoid cells and thylakoid stroma, along with degeneration of tubular epithelial vacuole granules. Additionally, focal brush border detachment and erythrocyte tubular pattern were visible in the tubular lumen, while no discernible lesions were detected in the renal interstitium or small arteries. The immunofluorescence analysis revealed negativity for IgA, IgG, IgM, C3, FRA, and C1q in four glomeruli. The pathological diagnosis indicated minor glomerular lesions, which require further examination by electron microscopy to exclude the possibility of hereditary renal disease. Linear α2(IV) chains were detected in the glomerular basement membrane, bursa, and tubular basement membrane using the renal basement membrane indirect immunofluorescence assay. On the other hand, α5(IV) chains were negative in all three areas-glomerular basement membrane, bursa, and tubular basement membrane. The electron microscope observation shows that there is mild segmental hyperplasia of the nuclear mesangial matrix in glomerular mesangial cells (Figure 1A). Additionally, there is an irregular thickness of the underlying membrane, localized dense layer, and alterations in layering. Furthermore, the majority of the foot processes of epithelial cells are fused, which indicates that the observation is consistent with AS.

The child was admitted to the hospital in December 2021 for treatment, receiving cefuroxime anti-infective, Lotensin 5 mg orally, astragalus granules for symptomatic relief, and methylprednisolone shock treatment for 3 days. The child’s general condition was satisfactory upon discharge with no specific complaints.

In 2022, the child exhibited a microalbumin/creatinine ratio of 457.28 mg/gcr, which increased to 540.21 mg/gcr in 2023 along with a 24-h urine protein quantification of 0.37 g/24 h. Urine red blood cell analysis revealed the presence of annular, crinkled, and anomalous erythrocytes in over 90.0% of full-field views, indicating elevated glomerulonephritic haematuria (Figure 1B).

### 3.2 Identification of *COL4A5* variant

**FIGURE 2:**
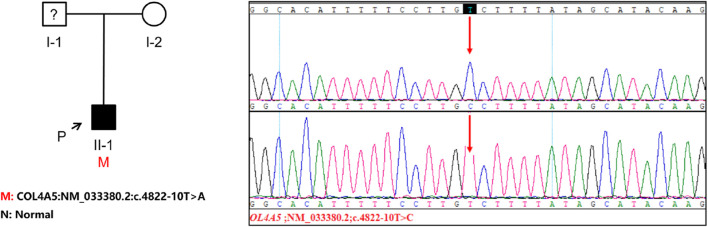
The investigation of familial lineage and sequencing of a patient hailing from a Chinese family. An arrow points to the patient involved in this study. The genomic DNA of both the patient and his mother was subjected to Sanger sequencing for analysis. The presence of a gene variation is visually indicated by a red arrow.

The genetic testing was carried out solely on the patient and the patient’s mother since the patient’s father refused to participate in the testing. The *COL4A5* gene in the patient was discovered to contain the c.4822–10T>C variant, which has not been documented in any literature and is absent from the gnomAD East Asian population database. It is worth mentioning that the patient’s parents show no indications of kidney disorder. To examine whether the mother also had the patient’s *COL4A5* gene variant, we conducted Sanger sequencing on both the patient and the patient’s mother. Our findings revealed that the mother did not possess the same variant, leading us to hypothesize that the variation was a novel occurrence (Figure 2). To analyze the variant c.4822–10T>C, we employed bioinformatics tools, namely, dbscSNV and SpliceAI. The Delta score (DS_AL) of 0.42 for SpliceAI acceptor loss indicates a potential impact on the process of clipping. The findings indicate that this genetic alteration has the capacity to induce abnormal splicing of *COL4A5* mRNA.

### 3.3 Confirmation of *COL4A5* variants as the factor for alternative splicing in splicing assay

**FIGURE 3:**
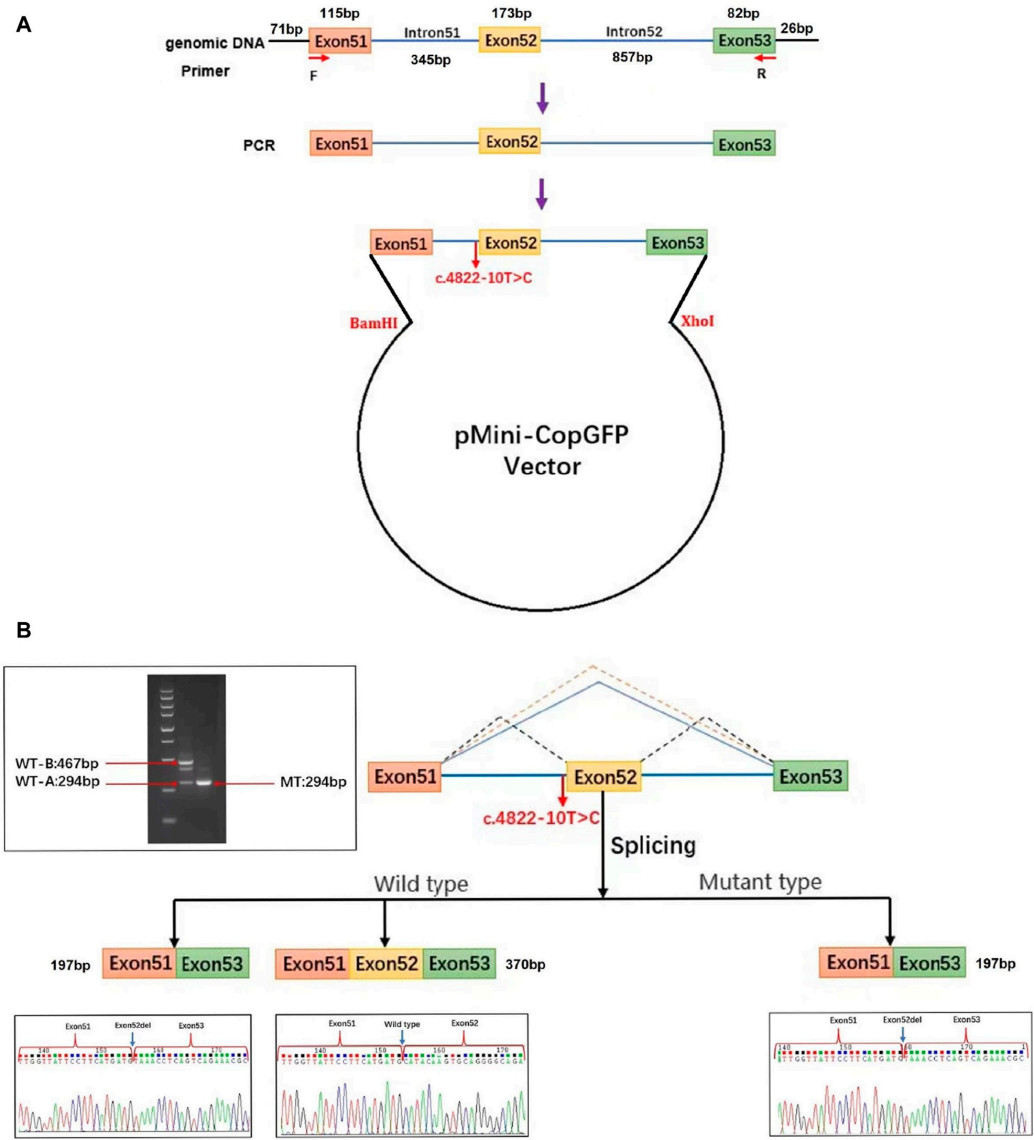
Analysis of exon deletion due to *COL4A5*: c.4822–10T>C splice variant. **(A)** Schematic illustration of the minigene pertaining to the *COL4A5* c.4822–10T>C variant. Exon 51 (orange), exon 52 (yellow), and a portion of exon 53 (green) along with adjacent introns (blue) of the COL4A5 gene were inserted into the pMini-CopGFP vector (black). The pMini-CopGFP vectors contained either wild-type (WT) or mutant (MT) sequences between the BamHI and XhoI sites. The positions of MiniRT-F and MiniRT-R primers, denoted as F and R respectively, were utilized for splicing analysis. HEK293T cells were transfected with the pMini-CopGFP vector containing WT or MT *COL4A5* hybrid minigenes. **(B)** Following RNA extraction and cDNA synthesis, the splicing products from the *COL4A5* minigene were amplified via PCR using MiniRT-F and MiniRT-R vector-specific primers, and subsequently visualized through agarose gel electrophoresis. Sanger sequencing confirmed the presence of a 467 bp WT-B splicing product as expected, along with an unexpected 294 bp WT-A product. The WT-B product comprised exon 51 (115 bp), exon 52 (173 bp), a portion of exon 53 (82 bp), and segments of the pMini-CopGFP vector (71 bp and 26 bp). In contrast, the WT-A product lacked the sequence of exon 52 present in WT-B. The MT splicing product of the COL4A5 minigene was a 294 bp fragment containing exon 51 (115 bp), a portion of exon 53 (82 bp), and segments of the pMini-CopGFP vector (71 bp and 26 bp). Notably, an intermediate product between 467 bp and 294 bp in size was identified in the WT splicing product of the *COL4A5* minigene. This intermediate product was further amplified through gel recovery, revealing six clonons upon clonal sequencing–three matching the WT-A sequence and three matching the WT-B sequence, with no other clonon sequences detected. Consequently, the middle band represented a mixture of WT-A and WT-B bands rather than a singular amplification product.

For this research, plasmids were designed to target the c.4822–10T>C variant of *COL4A5*, present in both the WT and MT forms. Afterwards, the plasmids were transfected into 293T cells (Figure 3A). The cells were used to extract RNA, which was then subjected to reverse transcription and followed by PCR amplification of cDNA. By examining the size of the PCR amplification products and the outcomes of Sanger sequencing, we verified the atypical splicing of the variant mRNA. Two mRNA products are generated by the WT plasmid. The first product is missing a 173 bp sequence from exon 52. This mRNA sequence was subjected to analysis using BLAST, revealing a predicted transcription product with similarity to the XM_016942897.2 transcript from the primate *Pan troglodytes*. The second product is as expected, containing the complete exon51, exon52, and exon53. However, the MT plasmid generates a single mRNA molecule, lacking a 173bp sequence in the mature mRNA, encompassing the entire exon 52. This mRNA is represented as NM_033380.2: c.4822_4994del (Figure 3B). The middle band of the WT plasmid was cut and recovered, followed by secondary amplification of the gel recovery product. The electrophoresis of the amplification product showed three bands. Subsequently, PCR products were cloned and sequenced, resulting in the detection of six clones. Among these clones, three matched the WT-A sequence and three matched the WT-B sequence, with no other clone sequences being identified. This led to the hypothesis that the middle band represented an electrophoretic mixture of two bands, WT-A and WT-B, rather than a single amplification product.

### 3.4 Analysis of abnormal structure and pathogenicity of *COL4A5* caused by variant

**FIGURE 4:**
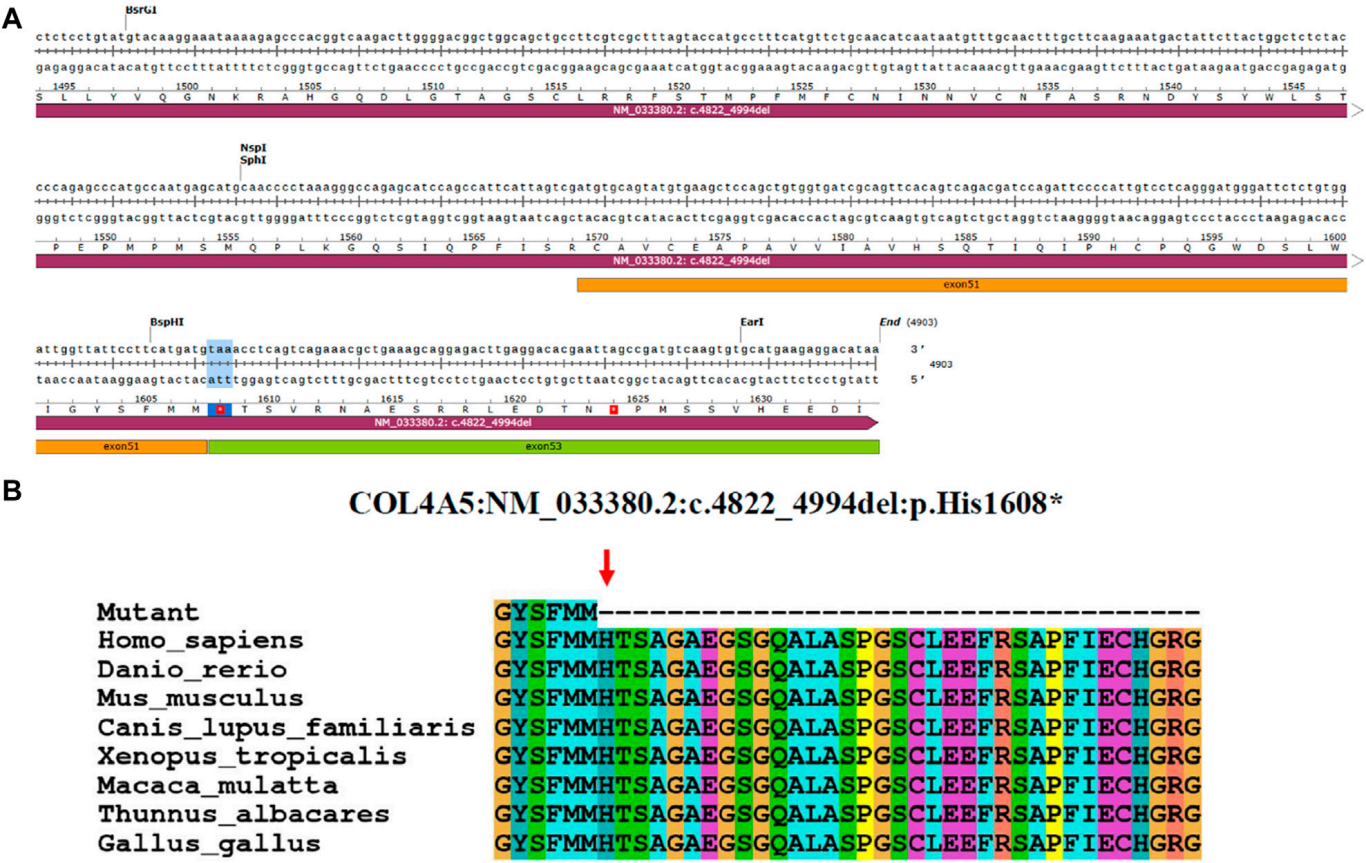
Analysis of *COL4A5* variants. **(A)** Base sequence changes in the *COL4A5* gene caused by aberrant splicing of c.4822–10T>C. **(B)** Evolutionary conservation of amino acid residues altered by p.His1608* across different species. NCBI accession numbers are *Homo sapiens*: NP_000486.1; *Danio rerio*: NP_001116702.1; *Mus musculus*: NP_001156627.1; *Canis lupus familiaris*: NP_001002979.1; *Xenopus tropicalis*: XP_004916922.2; *Macaca mulatta*: XP_014983488.2; *Thunnus albacares*: XP_044197271.1; *Gallus gallus*: XP_015134092.2. The amino acid terminus of the mutated protein is indicated by the red arrows.

**FIGURE 5:**
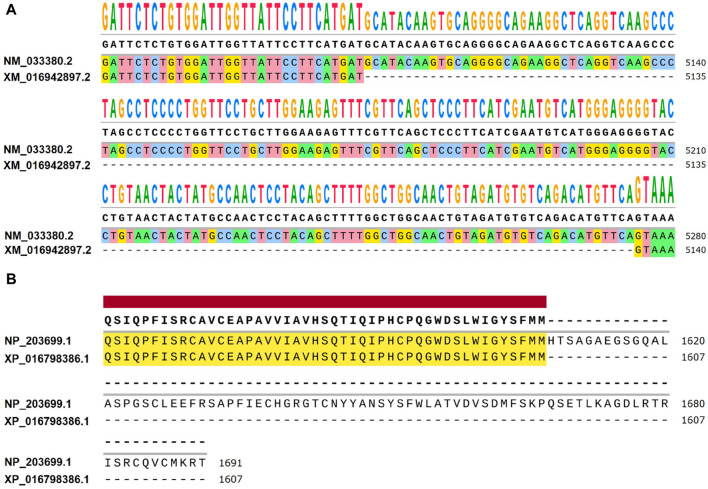
Differences between NM_033380.2 and XM_016942897.2 at the transcriptional and amino acid levels. **(A)** Differential expression levels are observed between *Homo sapiens* transcript NM_033380.2 and *Pan troglodytes* transcript XM_016942897.2. When comparing NM_033380.2 and XM_016942897.2, it is observed that the base sequence of exon 52 in the CDS region of NM_033380.2 is absent in XM_016942897.2, with notable differences in the UTR. **(B)** Differences in amino acid levels between *Homo sapiens* NP_203699.1 and *Pan troglodytes* XP_016798386.1. *Homo sapiens* NP_203699.1 has 1691 amino acids and *Pan troglodytes* XP_016798386.1 has 1607 amino acids.

The minigene experiments revealed that the *COL4A5* gene’s abnormal splicing variant c.4822–10T>C leads to a deletion of a 173bp sequence in exon 52, resulting in the mRNA NM_033380.2 c.4822_4994del. The c.4822–10T>C variant is characterized by a non-integer deletion, which induces a frame shift, ultimately culminating in the premature termination codon (Figure 4A). The initial three bases of exon 53 are consequently converted into the stop codon. When NMD is not present, a shortened protein is produced which does not include the section of the initial exon 53. This truncated protein is denoted as p.His1608*. After analyzing the evolutionary preservation of amino acid residues, it has been found that numerous amino acids before and after the human *COL4A5* protein H1608 show significant conservation in various species (Figure 4B). The NCBI blast comparison revealed the exon 52 deletion transcript of the *COL4A5* gene in primate samples, including *P. troglodytes* (XM_016942897.2), *Macaca mulatta* (XM_028841869.1), and *Chlorocebus sabaeus* (XM_007992531.2), while it was not detected in *Homo sapiens* (NM_033380.2) samples (Figures 5A,B). This observation raises the possibility of NMD occurring in this transcript in humans. As a result, we reevaluated the pathogenicity of the c.4822–10T>C variant following the ACMG guidelines. PVS1_Moderate: the aberrant splicing of c.4822–10T>C leads to the deletion of all exon 52 of the mRNA of the *COL4A5* gene, resulting in a 173 bp deletion. Conversely, the c.4822–10T>C variant represents a non-integer deletion that induces a codon shift, causing the first three bases of exon 53 to become a stop codon. Consequently, in the absence of NMD, the protein undergoes truncation, leading to the formation of a trun-cated protein; PP4: the clinical phenotypes exhibited a high degree of consistency with the single-gene he-reditary disease resulting from abnormalities in the *COL4A5* gene; PP3: the bioinformatics software forecasts the potential influence of the variant on gene splicing; PS3_Moderate: the functional analysis demonstrated that the variant had an impact on gene splicing, leading to the deletion of exons 52 and 53; PM2_Supporting: the variant is infrequent and has not been incorporated into the gnomAD database. Upon amalgamating the findings, it was determined that the base insertion resulting from the c.4822–10T>C variant was deemed to be pathogenic.

### 3.5 Analysis of the trimer α5(IV) and α345(IV) using 3-D structure examination

**FIGURE 6:**
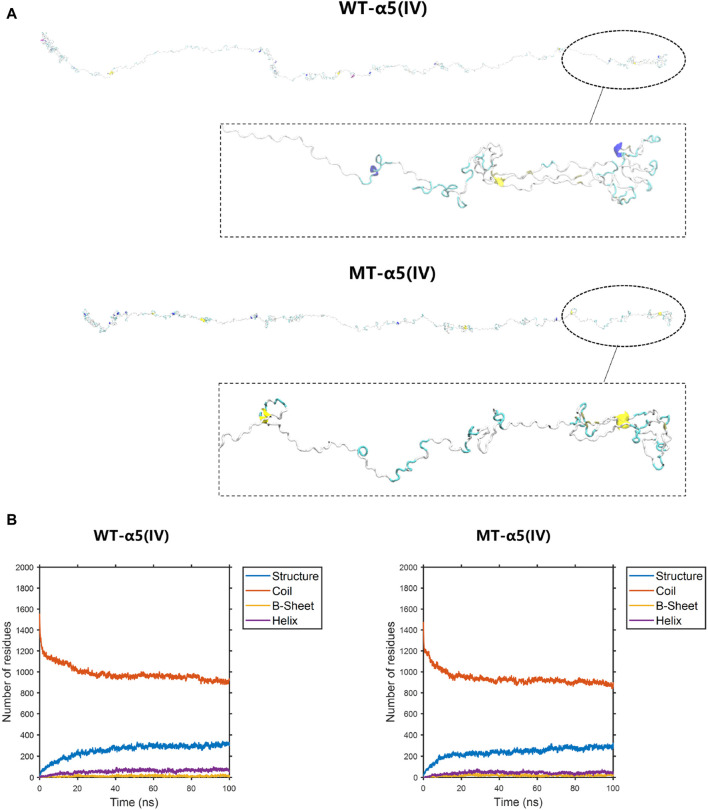
The conformation of the WT-α5(IV) and MT-α5(IV) chains. **(A)** The conformation of the WT-α5(IV) and MT-α5(IV) chains through molecular dynamics simulations at 100 ns. The formation of a truncated protein occurs because the MT-α5(IV) sequence is missing a total of 83 amino acids, starting from amino acid P1608, due to the existence of abnormal splicing c.4822–10T>C. **(B)** The numbers of the secondary structures of the WT-α5(IV) and MT-α5(IV) chains during 100 ns. After delete of “HTSAGAEGSGQALASPGSCLEEFRSAPFIECHGRGTCNYYANSYSFWLATVDVSDMFSKPQSETLKAGDLRTRISRCQVCMKRT” part in WT-α5(IV) chain, the number of the β-sheet in the MT-α5(IV) chain increased. Specifically, the number of β-sheet sturcture has increased from 18 to 25 and α-helix decreases from 56 to 44.

The construction of the model involved the utilization of the entire *COL4A5*: NM_033380.2 transcript (Figure 6A). The formation of a truncated protein occurs because the MT-α5(IV) sequence is missing a total of 83 amino acids, starting from amino acid P1608, due to the existence of abnormal splicing c.4822–10T>C (Figure 6A). As a result of this alteration, the quantity of β-sheets rises from 18 to 25, while the number of α-helices declines from 56 to 44 (Figure 6B).

**FIGURE 7:**
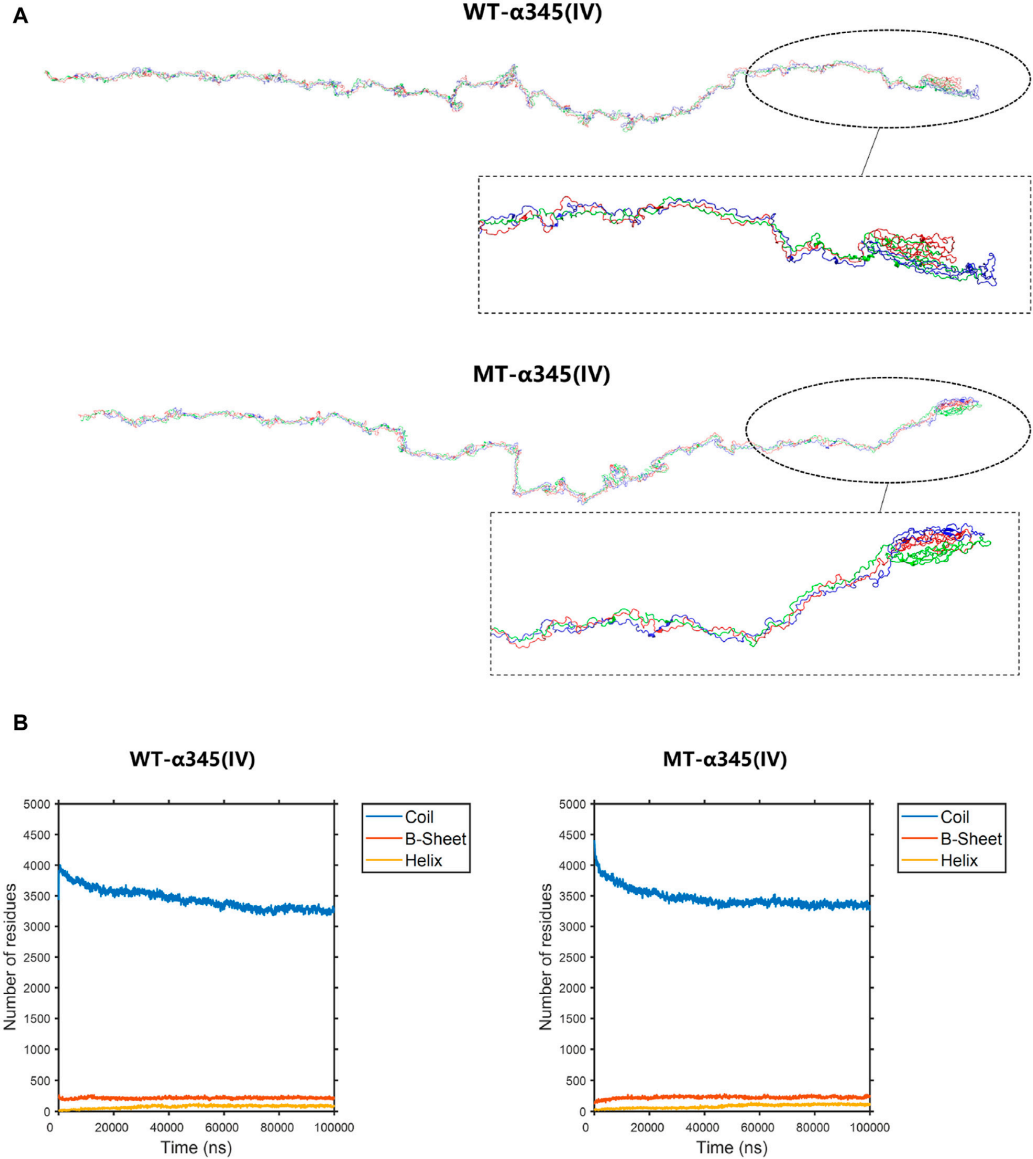
The conformation of the WT-α345(IV) and MT-α345(IV) trimers. **(A)** The conformation of the WT-α345(IV) and MT-α345(IV) trimers through molecular dynamics simulations at 100 ns. Following the p.His1608* variant, the MT-α345(IV) trimer is formed, maintaining a partially intact structure without complete collapse. **(B)** The numbers of the secondary structures of the WT-α345(IV) and MT-α345(IV) trimers during 100 ns. After delete of “HTSAGAEGSGQALASPGSCLEEFRSAPFIECHGRGTCNYYANSYSFWLATVDVSDMFSKPQSETLKAGDLRTRISRCQVCMKRT” part in WT-α345(IV) chain, the number of the β-sheet in the MT-α345(IV) chain increased. Specifically, the number of β-sheet sturcture has increased from 213 to 233 and α-helix increased from 85 to 107.

To display the three-dimensional arrangement of the WT-α345(IV) trimer, a ribbon-style presentation was employed (Figure 6A). Following the p.His1608* variant, the MT-α345(IV) trimer is formed, maintaining a partially intact structure without complete collapse (Figure 7A). An increase in the number of β-sheets and α-helices was observed when comparing the changes in secondary structure quantities between the WT-α345(IV) and MT-α345(IV) trimers. In particular, there was an increase in the quantity of β-sheet formations from 213 to 233, whereas the number of α-helices grew from 85 to 107 (Figure 7B).

### 3.6 Protein molecular dynamics simulation of the α5(IV) and α345(IV) trimer

**FIGURE 8:**
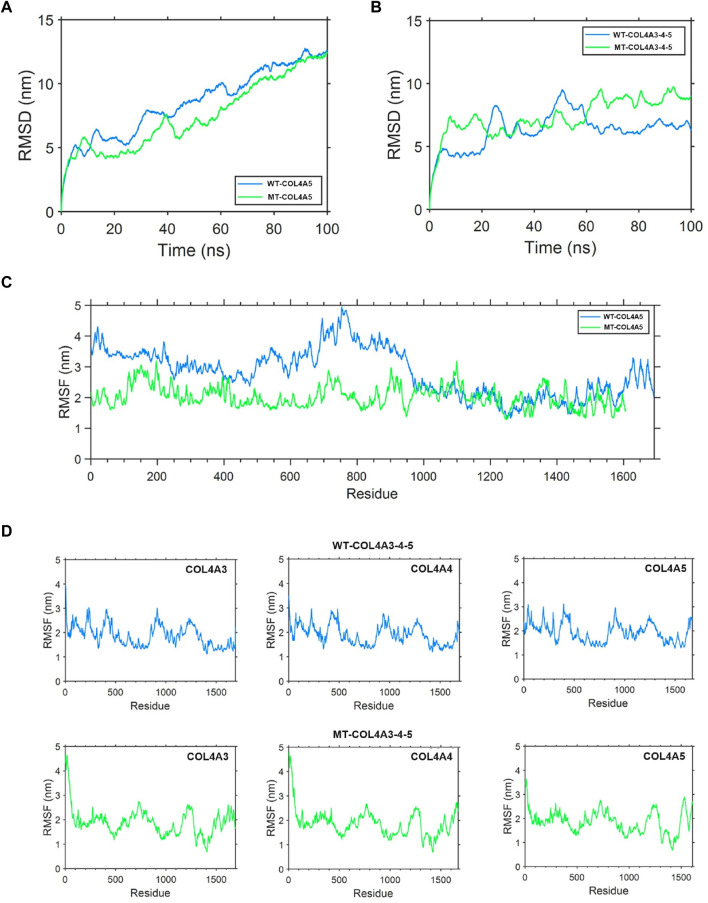
Protein molecular dynamics simulation of the α5(IV) chain and α345(IV) trimer. **(A)** RMSD of the WT-α5(IV) and MT-α5(IV) chains during 100 ns. The WT-α5(IV) chain reaches equilibrium quickly, in just 80 nanoseconds. The MT-α5(IV) chain requires a comparable duration of time (∼90 ns) to achieve a stable structure, owing to the absence of certain amino acid residues in the tail. **(B)** RMSD of the WT-α345(IV) and MT-α345(IV) trimers during 100 ns. The WT-α345(IV) achieves its stable configuration in a relatively brief period of 70 ns. Likewise, the MT-α345(IV) necessitates a similar timeframe of around 70 ns to attain a steady configuration, mainly due to an alteration in the amino acid at the end of the tail. **(C)** RMSF of each amino acid of the WT-α5(IV) and MT-α5(IV) chains from 80 ns to 90 ns. After analyzing the variations of amino acids in the MT- and WT-chains, it is clear that the WT-α5(IV) chain shows more instability towards the conclusion, whereas the MT-α5(IV) chain demonstrates reduced volatility because of the lack of specific amino acid residues. The head and middle regions of the MT-α5(IV) chain exhibit lower volatility than the WT-α5(IV) chain, indicating a lower level of activity in the MT-α5(IV) chain compared to the WT-α5(IV) chain. **(D)** RMSF of each amino acid of the WT-α345(IV) and MT-α345(IV) trimers from 80 ns to 90 ns. Significant differences were observed in the tail region of the MT-α345(IV) trimer when compared to the WT-α345(IV) trimer, as indicated by the findings of the RMSF analysis. This suggests that the MT-α345(IV) trimer has a relatively reduced stability in its tail, mainly due to the lack of a certain portion of the α5(IV) chain in the MT-α345(IV) trimer.

The root mean square deviation (RMSD) quantifies the degree of geometric disparity. According to the RMSD results, it can be observed that the WT-α5(IV) chain reaches equilibrium quickly, in just 80 nanoseconds. Conversely, the MT-α5(IV) chain requires a comparable duration of time (∼90 ns) to achieve a stable structure, owing to the absence of certain amino acid residues in the tail. The aforementioned observation indicates that genetic alterations have a minimal effect on the balance of the protein’s structural arrangement (Figure 8A). The root mean square fluctuation (RMSF) is utilized to quantify the deviation distance (in nm) of individual amino acid residues from their equilibrium positions during simulation. The magnitude of the peak reflects the degree of fluctuation exhibited by the corresponding amino acid residue. After analyzing the variations of amino acids in the MT- and WT-chains, it is clear that the WT-α5(IV) chain shows more instability towards the conclusion, whereas the MT-α5(IV) chain demonstrates reduced volatility because of the lack of specific amino acid residues. The head and middle regions of the MT-α5(IV) chain exhibit lower volatility than the WT-α5(IV) chain, indicating a lower level of activity in the MT-α5(IV) chain compared to the WT-α5(IV) chain (Figure 8C).

To examine the variation in geometric structure of the WT-α345(IV) and MT-α345(IV) trimers, a thorough molecular dynamics simulation was utilized to carry out a 100 ns kinetic analysis. The findings from the analysis of RMSD indicate that the WT-α345(IV) achieves its stable configuration in a relatively brief period of 70 ns. Likewise, the MT-α345(IV) necessitates a similar timeframe of around 70 ns to attain a steady configuration, mainly due to an alteration in the amino acid at the end of the tail. These findings suggest that variants exert minimal influence on the conformational equilibrium of the protein (Figure 8B). Significant differences were observed in the tail region of the MT-α345(IV) trimer when compared to the WT-α345(IV) trimer, as indicated by the findings of the RMSF analysis. This suggests that the MT-α345(IV) trimer has a relatively reduced stability in its tail, mainly due to the lack of a certain portion of the α5(IV) chain in the MT-α345(IV) trimer (Figure 8D). Additionally, it was observed that the variations in the head region of the MT-α345(IV) trimer displayed considerable size, suggesting that the MT-α5(IV) not only affects its trimerization process with WT-α3(IV) and WT-α4(IV), but also has an impact on the structural domain referred to as the “7S” at the beginning of the α345(IV) trimer (Figure 8D).

## 4 Discussion

A strong association between genotype and phenotype (Jais et al., 2000; Gross et al., 2002; Bekheirnia et al., 2010) exists in XLAS. According to Jais et al., people who have significant deletions and variants that cause premature termination of protein synthesis have a 90% probability of developing end-stage renal disease (ESRD) before reaching the age of 30. On the other hand, individuals with variants affecting the splicing sites face a 70% risk, while those with variants resulting in amino acid substitutions have a 50% risk (Jais et al., 2000). The individual being examined in this research displayed continuous blood in urine and slight presence of albumin in urine since the age of 5, without any associated hearing loss or eye abnormalities. Although the patient’s current condition appears to be mild, the early appearance of symptoms indicates a possibly severe phenotype. AS was confirmed by electron microscopic examination of the glomerular basement membrane, which revealed characteristic irregular thinning and thickening, along with diffuse basket weaving patterns. The *COL4A5* gene in this patient had a c.4822–10T>C variation that was not found in the gnomAD database. The parents of the patient showed no signs of kidney disease. The patient’s mother had a WT *COL4A5* gene and did not carry the same variant. Nevertheless, the father refused to undergo genetic testing, resulting in the unknown status of his *COL4A5* genotype. In order to evaluate the influence of the c.4822–10T>C variant on the main splicing locations, an analysis was performed utilizing different bioinformatics tools such as SpliceAI, dbscSNV_ADA, dbscSNV_RF, and varSEAK. The c.4822–10T>C variation is consistently associated with abnormal mRNA splicing, as indicated by multiple studies (Malone et al., 2017; Horinouchi et al., 2019). Through our *in vitro* experiments, we have ultimately verified that this alteration leads to the truncation of the protein. This determination will aid in the analysis of genotype-phenotypic correlation in the patient and facilitate future predictions regarding disease progression.

The GBM is composed of multiple triple helices, including α3(IV), α4(IV), and α5(IV), that join together. When there is a pathogenic variant in the encoding gene, causing a defect in one of these α chains, the GBM deteriorates, leading to the splitting of the lamina densa and resulting in the occurrence of the basket weave change. These modifications expedite the progression of glomerular sclerosis and result in impaired kidney function. The α5(IV) chains are affected by a pathogenic variant in the protein-coding gene *COL4A5*, leading to XLAS. This genetic alteration can result in two possibilities: either a total absence or reduction of the α5-chain protein, or a protein with amino acid replacement or addition that maintains its full length. The initial situation is simple, since an unfinished protein might not operate properly, leading to the emergence of illnesses. The addition or replacement of amino acids can cause localized bending or abnormal folding of the protein, which can affect the formation of the triple helix structure. Furthermore, protein molecules that are abnormally folded exhibit heightened sensitivity to proteases, rendering them more vulnerable to degradation (Kamura et al., 2020). Determining the severity of clinical phenotypes heavily relies on the importance of variants in the folding of the triple helix structure of the *COL4A5* gene. For this study, we employed molecular dynamics simulations to analyze how the c.4822–10T>C splicing variant of the *COL4A5* gene affects the ability of the α5(IV) chain to form a triple helix. Our goal was to gain more in-sight into the relationship between genotype and phenotype in individuals affected by this variant (Salomon-Ferrer et al., 2013).

The *COLA45* gene, also known as NM_033380.2, consists of a total of 53 exons. The sizes of the exons in *COL4A5*, excluding the untranslated sequences at the 5′and 3′ends, vary from 27 to 213 base pairs. Exon 1 consists of 283 base pairs, with 202 base pairs assigned to a 5′-noncoding sequence and 81 base pairs to a coding sequence. Exclusively, the signal peptide of 26 residues is encoded by the translated exon 1 sequence. Two Gly-X-Y triplets and 14 non-collagenous amino-terminal ends are encoded by exon 2. As a result, exons 2-47 contain the collagenous region, where exon 47 acts as a connecting exon responsible for encoding the carboxyl-terminal section of the collagenous region and a part of the non-collagenous region. The current study demonstrates that the c.4822–10T>C variant in the *COL4A5* gene causes the exclusion of exon 52 and exon 53 from its transcript, resulting in the production of a shortened α5(IV) chain. This truncated chain lacks a significant portion of the NC1 region sequence, which is crucial for trimerization. All-atom molecular dynamics simulations were used to perform a 100 ns kinetic simulation procedure on the MT-α5(IV) chain. According to our findings, the MT-α5(IV) chain displayed decreased variability in the head, middle, and tail sections in comparison to the WT-α5(IV) chain. This can be attributed to the removal of the tail. As a result, the MT-α5(IV) chain exhibited a lower overall activity compared to the WT-α5(IV) chain. Simulating the trimerisation of the MT-α5(IV) chain with the WT-α3(IV) chain and the WT-α4(IV) chain for a duration of 100 ns. According to the simulation findings, even with a partial removal in the NC1 area of the MT-α5(IV) chain, the theoretical possibility of trimerisation among the three chains remains. After undergoing trimerization, the MT-α345(IV) trimer displayed considerably more variability in its head and tail sections in comparison to the WT-α345(IV) trimer. This implies that the stability of the MT-α345(IV) trimer was decreased at the C- and N-terminal regions, possibly because of the impact of the MT-α5(IV) chain. Consequently, this had an effect on the overall stability of the fundamental structure of the BMs. Two potential scenarios may explain the lack of α5(IV) chain expression based on the patient’s renal pathology results. The initial situation includes the creation of a shortened α5(IV) chain caused by the c.4822–10T>C variant, which is then broken down by the protein quality assurance mechanism. The second scenario involves the production of an abnormal mRNA that is rapidly degraded by NMD, also resulting from the c.4822–10T>C variant. In either scenario, patients experience hindrance in the appropriate assembly of the α345(IV) trimer, thus requiring additional functional investigations to confirm this hypothesis.

## 5 Conclusion

To summarize, the splice variant c.4822–10T>C in the *COL4A5* gene was identified in a patient with XLAS. This expands the range of variants observed in the *COL4A5* gene, which follows an X-linked dominant pattern of inheritance. Histopathological examination of the patient’s kidney and minigene splicing validation experiments provided evidence for the pathogenicity of the variant, both *in vivo* and *in vitro*. Consequently, the findings of this investigation contribute to the advancement of our comprehension regarding the molecular pathogenesis of AS.

## Data Availability

The original contributions presented in the study are included in the article/Supplementary material, further inquiries can be directed to the corresponding author.
